# Integrating telemedicine in routine heart failure management: Experiences of healthcare professionals – A qualitative study

**DOI:** 10.1177/20552076241272570

**Published:** 2024-08-28

**Authors:** Jorna van Eijk, Jaap Trappenburg, Folkert W Asselbergs, Tiny Jaarsma

**Affiliations:** 1 Julius Center for Health Sciences and Primary Care, Department General Practice and Nursing Science, University Medical Center Utrecht, Utrecht, the Netherlands; 2 The Healthcare Innovation Center, Julius Center for Health Sciences and Primary Care, University Medical Center Utrecht, Utrecht University, Utrecht, the Netherlands; 3 522567 Amsterdam University Medical Centers, Department of Cardiology, University of Amsterdam, Amsterdam, the Netherlands; 4 Health Data Research UK and Institute of Health Informatics, 4919 University College London, London, UK; 5 123898 Department of Health, Medicine and Caring Science, Linköping University, Linköping, Sweden

**Keywords:** Telemonitoring, telemedicine, challenges in implementation, useability, professional expertise, heart failure, healthcare professionals, reflexive thematic analysis, qualitative study

## Abstract

**Objective:**

To describe the experiences of healthcare professionals with integrating telemedicine in routine heart failure (HF) care.

**Methods:**

Semi-structured interviews were conducted with healthcare professionals (*n* = 19) in the Netherlands who were involved in decision-making, implementation or routine use of telemedicine in HF management. Using purposive sampling, nurses, cardiologists and managers were selected to be interviewed. Interviews were performed in-person, recorded and transcribed verbatim. Interview data were analysed using a reflexive thematic analysis.

**Results:**

This study identified four themes: (1) Responsibility – the lack of a clear delineation of roles and responsibilities among healthcare professionals, patients and suppliers in telemedicine. (2) Confidence and safety – telemedicine is seen by healthcare professionals as capable of enhancing safety, yet also introduces the risk of fostering a false sense of security among patients. (3) Collaboration – actively involving end-users in the development and implementation of telemedicine promotes the adoption. (4) Processes and mutual agreements – rather than replacing traditional care, telemedicine is perceived as an adjunct to it. Structured care pathways support telemedicine implementation, and personalised telemedicine can empower patients in self-care.

**Conclusions:**

Telemedicine is a promising intervention in the management of HF. However, existing systems and care pathways have resulted in limited adoption. Improvements in the collaboration and establishing clear agreements on responsibilities between professional, patient and supplier can lead to more confidence in adopting telemedicine. Structured care pathways can be supportive. A personalised telemedicine approach can ensure that telemedicine remains manageable for patient and professional.

## Introduction

With advancements in science and technology, telemedicine technologies have evolved rapidly towards a multi-component intervention increasingly integrated in routine clinical care.^1^ Telemedicine is defined as ‘the exchange of health information and/or care instructions using digital techniques, where patient and provider are separated by distance, to allow and optimise the process of care remotely’.^2^ Drivers to implement telemedicine are the increased number of patients with chronic diseases, organisational burden (i.e. high healthcare costs and shortage of healthcare professionals) and the need for patient empowerment.^3^

In patients with heart failure (HF), telemedicine is used as a tool to optimise HF management, self-care support and symptom monitoring to improve care and prevent hospital (re)admissions.^4^ HF is a clinical syndrome associated with an unpredictable but in general progressive disease trajectory.^4^ Numerous studies evaluated the (cost-) effectiveness of telemedicine in HF care trajectories. These studies hint towards a positive effect on hospital (re)admissions, mortality and costs.^5–7^ Health insurance companies and patient organisation are advocating for the expedited adoption of remote HF management, while HF guidelines are cautious in prescribing telemedicine.^3^ Hence, the implementation and integration of telemedicine varies substantially across different hospitals and countries. Patient and healthcare professionals in general seem satisfied with using telemedicine in clinical practice, but they also encounter challenges and barriers.^8–13^

Despite the positive results of telemedicine on health outcomes, and the lobby by health insurances, patient organisations and telemedicine suppliers, only a minority of the patients are provided with remote HF management.^14^ A recent study using healthcare claim data from the largest Dutch insurance company shows that a third of the Dutch hospitals had telemonitoring claims. Within these hospitals, 6% of patients who were treated for HF received telemonitoring.^14^ To improve the integration of telemedicine in HF care, a first step is to understand why only a minority of the patients are provided with remote HF management by nurses and cardiologists, while the tools are available. Hence, in addition to theoretical reflection, it is valuable to seek insights from professionals in the clinical practice. Therefore, this study aimed to investigate the experiences of healthcare professionals with integrating telemedicine in routine HF care.

## Methods

A qualitative, reflexive thematic design with an inductive analysis according to Braun & Clarke was used to explore experiences of healthcare profession, in the integration of telemedicine in HF care using semi-structured interviews.^15,16^ This study followed the COnsolidated criteria for REporting Qualitative research (COREQ) (Supplemental file 1: COREQ Checklist).^17^ The Medical Ethics Research committee of the University Medical Center Utrecht, the Netherlands, approved this study (approval number: 22/497).

### Participants and setting

The current study is part of the Responsible roll-out of e-health trough systematic evaluation in Heart Failure (RELEASE-HF) study, a study about the effectiveness of telemedicine among patient subgroups with HF, that collects data on patient characteristics, health outcomes and telemedicine applications.^18^ Eligibility criteria included experience with performing telemedicine in patients with HF and/or involved in the process of decision making on implementation of telemedicine in their hospital, working in an academic or non-academic hospital in the Netherlands and willing to participate. Using a purposive sampling strategy, contact persons of the RELEASE-HF hospitals who offer telemedicine received an information letter by email and were invited to participate in an interview study about their telemedicine intervention, motives and challenges they face. Additional participants who did not work in a RELEASE-HF hospital but offer telemedicine in the HF outpatient clinic were approached by phone and received the information letter by e-mail.

Interviews were conducted with 19 participants (Table 1). The participants were employed as a nurse (11/19, 58%), manager with a focus on digital health and/or cardiology (6/19, 32%), or cardiologists (4/19, 21%). Participants had between 2 and 12 years of experience in telemedicine. Four (19%) participants did not work in patient care. The majority of participants were affiliated to a non-academic hospital in the Netherlands.

**Table 1.** Characteristics of participants.

Characteristic	Participants (*n* = 19)
Age, years (mean (SD))	49.7 (11.6)
Female (*n* (%))	13 (68)
Function (*n* (%))^a^	
Heart failure nurse	8 (42)
Nurse practitioner	3 (16)
Cardiologist	4 (21)
Manager cardiology	4 (21)
Consultant innovation and eHealth	2 (11)
Type of hospital (*n* (%))	
Academic	5 (26)
Non-academic	14 (74)
Telemedicine status in hospital (*n* (%))	
User	16 (84)
Starter	2 (11)
Recently stopped	1 (5)
Years of telemedicine experience (mean (SD))	5.7 (4.1)

^a^ Sum of categories in ‘Function’ is more than the total number of participants, due to participants with dual functions.

### Data collection

Semi-structured interviews with closed- and open-ended questions were conducted using an interview guide. Interview guides were developed to explore the providers’ experiences towards challenges and opportunities on various aspects of telemedicine. The interview guides were based on literature, developed with multiple professionals with expertise in nursing care, telemedicine, research and HF care, and validated by external experts. We designed two different interview guides, using telemedicine taxonomies.^19,20^ Because of the mixture of participants, we developed an interview guide for healthcare professionals with experience in working with telemedicine and another one for managers (Supplemental file 2: interview guide). These guides differed in topics about in-depth details of the telemedicine intervention, the practical use and experience in patient care, implementation, policy and financial regulations. An overview of the interview topics can be found in Table 2.

**Table 2.** Interview topics about telemedicine integration in HF management.

**Interview topics**
*Healthcare professionals using telemedicine*
- Aim of telemedicine in HF management - Provision of telemedicine Patient characteristics Members of the HF team and characteristics - Telemedicine system Supplier Features (telemonitoring, tele-education, communication, self-management) Devices - Evaluation: expectations, experiences - Time investment ***-*** Future perspectives: recommendations and improvements
*Managers*
- Aim of telemedicine in HF management - Telemedicine supplier (choices, reasons, arrangements) - Organisation of care (vision, motives, structure of the outpatient clinic, HF teams) - Implementation and prerequisites - Health insurance company - Evaluation, expectations, cost-effectiveness - Collaboration with primary care ***-*** Future perspectives: recommendations and improvements

Interviews lasted 60–90 min and were conducted in person at the workplace, in a private room. Interviews were only held virtually via Microsoft Teams in case of practical reasons of the participant. Interviews were conducted by JE (female; experienced in interviewing) and in half of the interviews a student from the master Nursing science (JH) (male) assisted. All interviews were performed between April and October 2022, and completed based on saturation, that is, when additional interviews did not lead to new themes or perspectives.^21^ Participants signed informed consent and were informed that the interviews would be audio recorded, transcribed verbatim and pseudo-analysed.

### Data analysis

**Figure 1.**
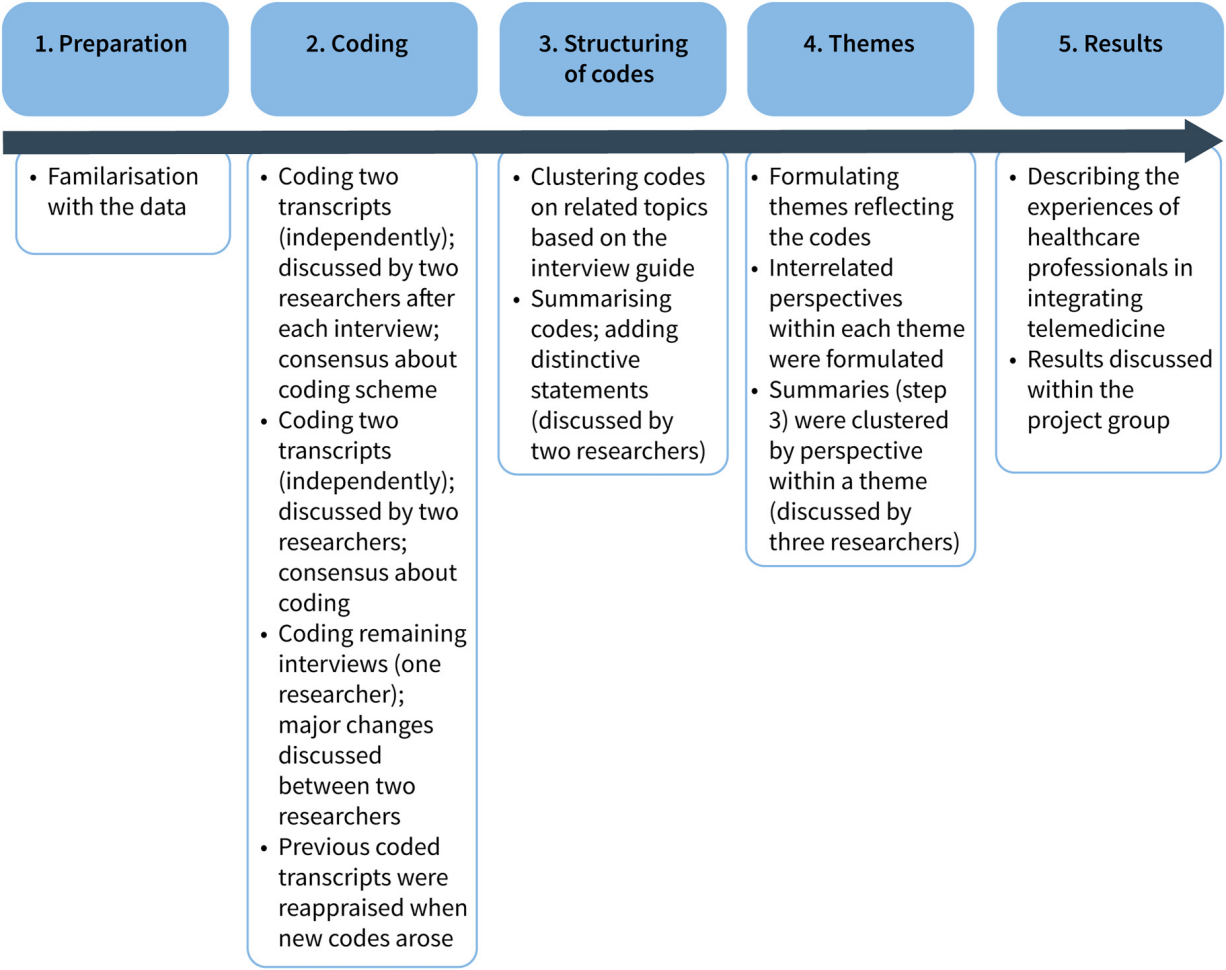
Steps performed in the qualitative analyses.

Reflexive, thematic analysis of Braun and Clarke was used to analyse the transcripts and coding was performed using NVIVO version 12 (QRS International).^15,16^ All steps and decisions were written in an audit trail. Figure 1 summarises the steps of data analysis. After familiarisation with the data by reading the transcripts, initial codes (codes are parts that describe the content of the phrase) were generated by two researchers (JE and TJ) independently. Both interviewers had been trained in qualitative research, are experienced, and have a background in nursing. Four interviews with different perspectives were coded by both researchers to ensure consistency and rigour.^22^ After coding the first interview, and after coding the second interview, the researchers discussed emerging codes until consensus was reached. Next, the third and fourth interviews were coded independently and codes were discussed afterwards until consensus was reached. Subsequently, one researcher (JE) coded the remaining thirteen interviews, and discussed these transcripts with the other researcher (TJ) when major changes in coding were required. When new codes were added previous coded transcripts were checked for the new codes. After the coding phase, all codes were analysed and structured on related topics based on the interview guide (Supplemental file 3: clustering of codes). Subsequently, these codes were summarised and distinctive statements were added. These summaries were discussed by two researchers (JE and TJ) and themes reflecting the data were reviewed (themes are overarching, recurring topics that emerge from the data after a process of coding and analysis). Based on these discussions, we noticed that within each theme three interrelated perspectives play a pivotal role: the organisation (i.e. hospital, healthcare professional, and outpatient clinic), patient and their relatives, and the telemedicine intervention (i.e. supplier, features of the intervention). Next, the structuring on related topics was then detached, and summaries were assigned per theme and their perspectives. This was discussed with a third researcher (JT). Finally, the experiences of healthcare professionals towards the challenges and opportunities of the integration of telemedicine in routine HF care were described per theme. These descriptions were discussed within the project group until consensus about the content of the themes was reached.

### Setting: telemedicine characteristics

The telemedicine interventions that the participants used include telemonitoring and tele-education. Within telemonitoring, monitoring and transfer of physiological data (weight, blood pressure, heart rate) are included. Depending on the telemedicine system, automatic questionnaires on HF symptoms are collected standard or incidentally. Some telemonitoring systems work with Bluetooth connected devices (e.g. weight scale). Tele-education consists of educational modules explaining HF, how to signal deterioration, medication and self-management. Some systems provide self-test questions. All telemedicine interventions have an online environment for communication between patient and provider, but the way of contact is not always two-sided (i.e. from patient to provider and vice versa). Some hospitals collaborate with a supporting centre which is an (external) centre that provides support for nurses at the outpatient clinic, handling the data and messages that patients sent via the telemedicine intervention.

## Results

**Figure 2.**
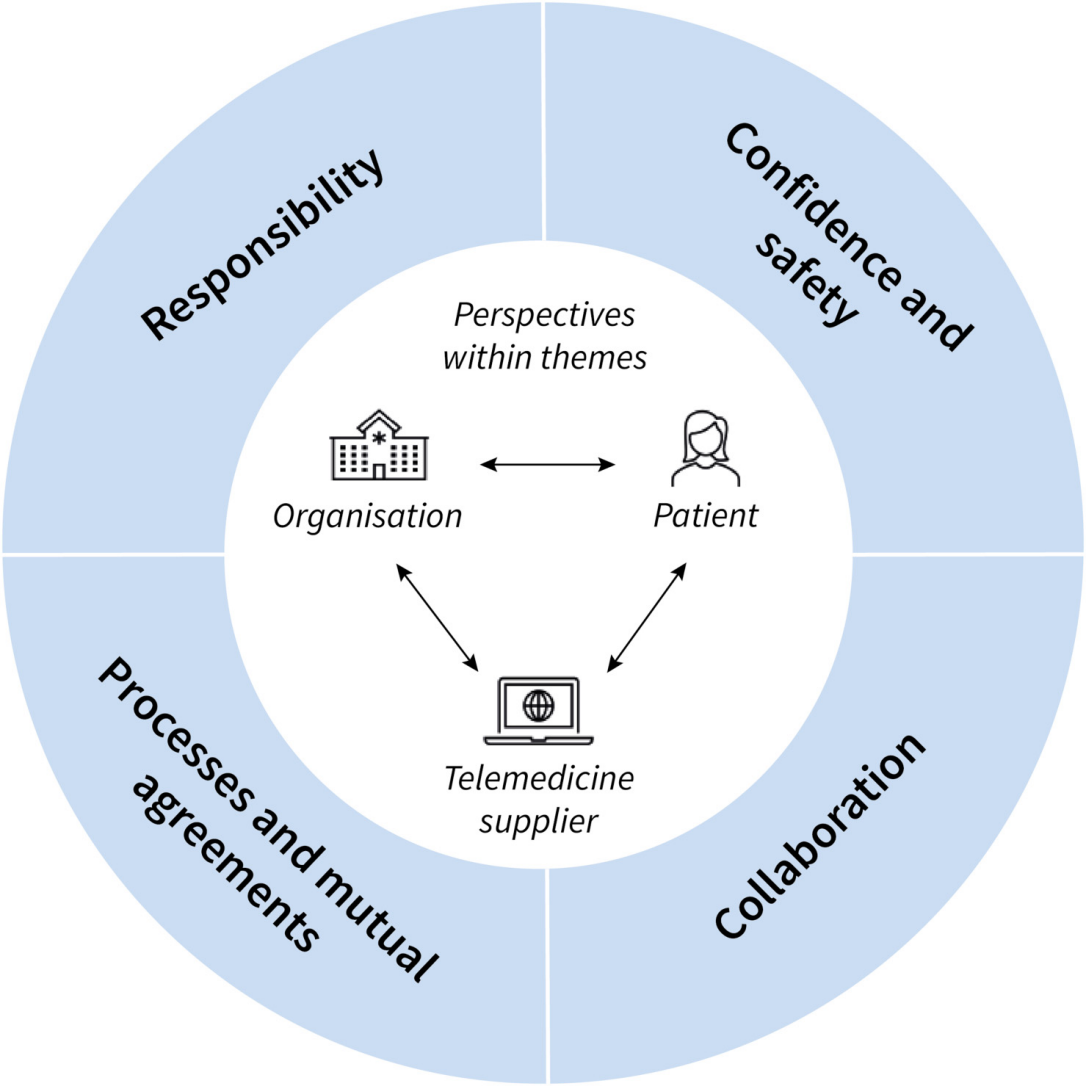
Schematic overview of the four themes – responsibility, confidence and safety, collaboration, workflow – which reflects the challenges and opportunities healthcare professionals experience towards the integration of telemedicine in heart failure care. Within each theme, three interrelated perspectives play a pivotal role: the organisation (hospital, healthcare professionals, heart failure outpatient clinic), the telemedicine supplier (supplier, components and features of the system), and the patient (patient with heart failure living at home). These perspectives are important elements in the uptake of telemedicine in daily practice.

Four overarching themes emerged from the interviews: (1) responsibility, (2) confidence and safety, (3) collaboration, and (4) processes and mutual agreements. Figure 2 visualises these themes and their relation to the three interrelated perspectives within each theme: organisation, patient, telemedicine supplier.

### Theme 1: responsibility

Healthcare professionals reported feeling highly responsible for their patient's health but the implementation of telemedicine added challenges regarding the extent to which they should be held accountable for the patients’ actions remotely. Establishing clear agreements on responsibilities among healthcare professionals, patients, telemedicine supplier and a supporting centre is needed for successful integration of telemedicine. In this theme, the following subthemes are included: self-management as a driver for responsibility, shared responsibility, and professional responsibility.

#### Self-management as a driver for responsibility

Participant’s emphasise that one of the main objectives for implementing telemedicine in HF care is to promote self-management. Supporting self-management could enhance patients’ sense of responsibility for their own disease trajectory and support patients in actively participating in the management of their disease. A participant expressed this as patients being an equal partner in HF management. Conversely, professionals also express concerns that telemedicine might encourage passive behaviour if patients overly rely on the advises and actions of healthcare professionals based on the telemonitoring readings.

People do have a strong tendency to put the responsibility outside themselves. Like, I’m being supervised at the HF outpatient clinic [by a HF nurse], so you’re responsible for me. (Nurse practitioner)

Furthermore, the telemonitoring frequency and notifications result in patients being confronted by their disease on a daily basis, resulting to a reluctance of some patients to use telemedicine. In contrast, certain professionals note that younger HF patients particularly express that telemedicine provides them with the opportunity to better manage their disease and enhances their ability to enjoy life.

#### Shared responsibility

Participants expressed varied opinions on who is responsible for the healthcare and disease management of patients with HF: the patient, the healthcare professional, a supporting centre, or the telemedicine supplier. The relationship between the patient and the healthcare professional is important in determining who bears the responsibility for the patients’ health.

If you don’t give responsibility to the patient, then the patient will not t take it. Therefore, in that context, I believe there is a trade-off. (Cardiologist)

However, discussions about responsibility in healthcare arise, patients are responsible for their own health and how to manage their disease versus the healthcare professional is responsible for the health of the patient. Some healthcare professionals emphasise that patients bear responsibility for deciding whether to utilise the telemedicine intervention and, if they do, for ensuring its proper use so professionals can treat them. An example of incorrect use of telemedicine observed was a patient providing favourable responses to questionnaires regarding HF symptoms, leading to misinformation and ambiguity for the professional. These favourable answers were sometimes given when patients dislike the questionnaires due to having to fill them out too often.

You cannot force them [to use telemedicine]. We really try to encourage patients, but if someone does not want to anymore, then it stops. I don’t continue to engage with people who really don’t want to.(HF nurse)

However, others argue that the responsibility lies with nurses or physicians to carefully assess whether a patient has sufficient competence to take control of their disease trajectory, regardless of whether it includes telemedicine or not.

#### Professional responsibility

Some participants shared the passion that nurses have for their work can lead to an increased feeling of responsibility. They perform more tasks than what is expected in the use of telemedicine, such as calling patients when they exceed thresholds instead of waiting for the patient to call. Some also continue planning regular outpatient clinic visits alongside telemedicine monitoring, rather than reducing these visits. Such a lack of clear boundaries can lead to an increased workload. At the same time, participants explained that professionals were reluctant to delegate care to their patients with chance of deterioration, due to an inner struggle of losing control over your patient versus extending professional responsibility. Telemedicine requires a change in how healthcare is provided, but some participants perceive it as *undermining* their profession.

When we started with telemedicine then the HF nurses were like: Oh, but aren’t we needed anymore? But personal counseling is of course very different from telemedicine. Telemedicine is just an extra way of care. (Nurse practitioner)

Before the implementation of telemedicine, nurses lacked the capability to monitor patients at home, even though patients routinely conducted measurements themselves, for example, weight or blood pressure. One participant emphasises that the healthcare professionals did not feel this absence of continuous access to data as a problematic. However, with the implementation of telemedicine, concerns about responsibility and the necessity for clear agreements have become apparent. Subsequently, some healthcare professionals express concerns regarding who is liable when complications arise in patients who receive remote HF management. They are wondering whether they can be held responsible for the treatment and well-being of their patients when it involves telemedicine and when patients are monitored by a supporting centre. In the current care, there is a lack of clear guidelines on the accountability in telemedicine.

Healthcare professionals are concerned if we no longer see the patient in-person [at the outpatient clinic], can I still be responsible for the patient and can I give proper care? (Manager)

### Theme 2: confidence and safety

Telemedicine leads to changes in the organisation of care, that is, fewer physical visits to the outpatient HF clinic and more digital interactions between patient and nurse. A key aspect in using telemedicine is the confidence that both patients and healthcare professionals have in the usefulness and safety of the telemedicine intervention. In this theme, the following subthemes are included: confidence of healthcare professionals, conflicting feelings of safety, and competence of patients.

#### Confidence of healthcare professionals in telemedicine

Digital contact was experienced by the participants as limiting their clinical judgement which was felt as an important part of their profession. The same was experienced when outpatient clinics collaborate with a supporting centre. Most of the participants have positive experiences with these centres, such as reducing workload, but some professionals experience that they have less insight in the status of their patients. Relying on data collected outside their direct reach and relying on a supporting centre makes them feel less confident but also raises doubts on maintaining safety. As a solution, some participants set thresholds in the telemedicine intervention tight, so they receive a notification in an early phase of a possible deterioration, resulting in a higher workload.

If there are notifications [exceeding thresholds] and the patient didn’t call this morning, but based on the measurements I think that something isn’t right, I’ll still call the patient. (HF nurse)

#### Conflicting feelings of safety

Participants indicate that telemedicine contributes to safety by facilitating easy contact between patient and professional, enabling earlier detection of deterioration, and even identifying previously undetected problems (e.g. arrythmia). Telemonitoring was also perceived as adding value by safely facilitating titration of drugs more rapidly. However, it was also seen to potentially instil a false sense of safety in patients. Patients think that when they use telemedicine someone monitors them for 24/7, but actually the healthcare professionals only monitor the patient at specific moments during the day. Additionally, monitoring is mostly lacking during out of office hours according to the professionals. When patients have fewer capabilities to manage their own disease and thus wait for a professional to contact them, because they think they are monitored 24/7, this could result in preventable deterioration. This makes healthcare professionals doubt whether to provide telemedicine to all patients.

#### Competence of patients

Participants reported occasional doubts regarding a patient's competency to perform the telemonitoring. Some nurses experience that a patient's lack of digital skills is associated with older age but others mention that age is not a limiting factor to use digital technology. Participants described that especially older patients wish to visit a cardiologist or nurse in-person, whereas younger patients have more confidence in and are willingness to use telemedicine to manage their disease.

We are actually just, for our feeling, five years too early in this [using telemedicine] system given our current patient population. (HF nurse)

Healthcare professionals have observed that patients seek confirmation regarding the accurate monitoring of their vital signs and often require encouragement from professionals to manage their disease effectively. Conversely, professionals find telemonitoring to be a valuable tool in empowering patients with confidence and control over their disease management. One participant suggests that a supporting centre could further bolster this sense of confidence by providing easily accessible contact. However, there is also recognition that this additional layer of healthcare may inadvertently undermine patient confidence, especially if they previously had direct contact with nurses and now face an added layer of interaction.

I’m convinced that telemonitoring is really good for patients, especially for patients with chronic diseases who feel something every day which could make them insecure. Telemonitoring is a really good way to get certainty, feel safe and gain insight into your disease. (Cardiologist)

### Theme 3: collaboration

Collaboration involving regular evaluations of the design, technical features, and lay-out of the telemedicine tool among healthcare professionals, patients, and the telemedicine supplier is considered as important for successful integration of telemedicine in routine HF management. We identified the following forms of collaboration: healthcare professionals and supplier, interprofessional collaboration, and collaboration between patient, healthcare professional and telemedicine supplier.

#### Collaboration between healthcare professionals and telemedicine suppliers

Healthcare professionals mention the necessity of telemedicine intervention to be more user-friendly. They struggle with unclear overview of patient measurements, difficulties in setting thresholds, notifications sometimes lacking clarity in urgency, and the lack of integration with electronic health records (EHR). Some participants have existing collaborations with suppliers, which gave them control over the content of the telemedicine intervention. Others aspire to establish such partnerships. However, healthcare professionals perceived that since telemedicine suppliers have expanded into larger companies this hampered the ease of collaboration due to less personal contact.

#### Interprofessional collaboration

Motives for using telemedicine differ within teams, that is, not all professionals are in favour for remote HF management. Contradictory study results and the lack of direction in HF guidelines are main arguments for not using telemedicine.

My colleagues are a bit wait-and-see. We have to experience what it will bring. They are not very enthusiastic right away. But then I have to admit that ten years ago I wasn’t either. I also had questions about if telemedicine is good intervention in HF and how are we going to integrate telemedicine in our daily work? It's a change after all. So it's a bit of logical cold feet. (Nurse practitioner)

Managers express that the intrinsic motivation of healthcare professionals is key for the successful integration of remote HF management. They explain that the integration of telemedicine should never be a top down decision. Having a *champion* who is also a healthcare professional can support implementation and it is supportive to perform regular evaluations about the experience of telemedicine in HF management with members of the HF team.

#### Patient, healthcare professional and telemedicine supplier

Patients’ needs can diverge from those from professionals and suppliers in aspects as: the design of the intervention, technical features, educational topics, frequency of measurements and notifications. The participants advocate that patient involvement in the design of the intervention leads to a better fit with patients’ need, resulting in an increased use of the intervention and better self-management. An example to express these misalignments in needs and expectations is the incorporation of a mandatory quiz after finishing an educational module. For healthcare professionals, the quiz is useful to check whether a patient understands the information. But for patients these quizzes could lead to frustration and reluctance to use telemedicine.

If I tell you: “you are diagnosed with HF”, in two minutes you have already searched on the internet what this diagnosis means, how it works, and how to manage your disease. That is the generation of patients coming up. For a telemedicine supplier, the challenges are to anticipate on this behavior. Which means to stop being supply-driven and work much more demand-driven. (Manager)

### Theme 4: processes and mutual agreements

Structured procedures and mutual agreements within the hospital between departments and/or healthcare teams (members), as well between hospitals and primary care about telemedicine, support the integration of remote HF management, according to the participants. Creating structured processes is complex because it requires alignment various levels within the healthcare process. We identified three levels of processes: (1) at the macro level between hospitals and primary care, (2) at the meso level with structured care paths within the organisation, and (3) at the micro level the features of telemedicine intervention when it is integrated in HF care.

#### Macro level: continuation of telemedicine outside the hospital

Some healthcare professionals are critical about implementing telemedicine in HF management, because when they refer a stable and/or non-complex HF patient to the general practice (GP) this patient cannot continue with telemedicine. In the Netherlands, telemedicine for HF care is not yet implemented in primary care, and reimbursement in primary care by health insurances is not yet established. However, the participants indicate that for the continuation of care remote HF management is also important in primary care. Some hospitals set-up regional collaborations to collaborate with primary care, which are evaluated as positive.

Much more collaboration with primary care is needed. We need the concept of the ‘hospital without walls’. Nowadays, it's still very much inward-looking. (Nurse practitioner)

#### Meso level: challenges in integrating telemedicine in imperfect care pathways

Some participants do not see the added value of telemedicine since care pathways (i.e. when to start telemedicine, who receives telemedicine, who is involved, what changes should be made in the structure of the outpatient clinic, how is the reimbursement) are not clear. Substitution of care is one of the goals for implementing telemedicine in HF management, however the participants argue that this is mostly lacking. Additionally, they criticise the cost-effectiveness of telemedicine, because in their opinion there is only a shift of costs in the outpatient clinic instead of a reduction (i.e. telemedicine leads to fewer outpatient clinic visits, therefore a hospital can save on personnel costs. However, telemedicine also requires continuous monitoring, necessitating more nurses to control the notifications). The healthcare professionals perceive a contradictory effect of integrating telemedicine into the outpatient clinic. On the one hand, it decreases the amount of patients in the outpatient clinic, shortening outpatient appointments through prior information provided via the telemedicine tool about the patient's current situation. This leads to a perceived reduction in workload. On the other hand, telemedicine increases the outpatient clinic's burden as more severe patients visit and despite a shortening outpatient appointment they need more preparation time. The amount of incoming data and messages from the telemedicine system further increases the workload. A balance is sought in how much extra work telemedicine gives versus patient satisfaction when using telemedicine.

At the moment it is still very much on top of care as usual. We do both outpatient visits and we call patients and we provide telemedicine. But in the long run you also want that telemedicine reduce the workload. (HF nurse)

However, in some hospitals they are very strict in reaching substitution of care.

This is communicated very clearly from the board: either we stop with telemedicine or we stop with outpatient visits. We are not going to do extra work because you simply don’t have time for that either. (Manager)

According to the participants, more attention is needed for the embedding of telemedicine in their routine care pathway, otherwise the telemonitoring will only increase instead of reducing the workload. Another remarkable aspect is that some hospitals have an agreement with the telemedicine supplier, including a license specifying a maximum number of users allowed to access telemedicine. This limitation hinders the number of patients using this intervention and substituting care.

#### Micro level: limitations and room for improvement in the features of telemedicine

Many telemedicine interventions lack personalised advise (i.e. personalised education modules, medication advises, self-management actions, notifications) which could be supportive according to professionals. Main features of the current telemedicine applications are monitoring vital signs, sending messages and alarming healthcare professionals when thresholds are exceeded. Participants observe that an excessive number of notifications adversely affect patients’ use of telemedicine. However, some professionals are hesitant about whether automated personalised messaging for patients is effective and desirable. It could improve self-management, but for the professionals it gives a sense of losing control over the patient.

I do find it [automatic generated personalised advises] very tricky. Because normally you always have to ask the patient questions about complaints. And if the system [telemedicine intervention] doesn’t do that for me, then the system can draw the wrong conclusion. As a results, I end up with a hospital admission that I wouldn’t have wanted. (HF nurse)

I think it's good for some people to be in control of their own affairs. I think it gives them a certain satisfaction if they manage their disease well. That they [the patients] feel like: I don’t have to bother anyone about it and I’ll solve it myself. (HF nurse)

## Discussion

We set out to describe experiences of healthcare professionals towards the integration of telemedicine in HF management in four main themes: responsibility, confidence and safety, collaboration, and processes and mutual agreements. These findings help us to understand why a minority of patients at the HF outpatient clinic receive or use telemedicine. Motives to integrate telemedicine in HF management include its perceived usefulness during titration, promotion self-management, reduction of outpatient visits and duration of consults, early detection of deterioration, and increased patient involvement in their own healthcare. However, substantial challenges were experienced when integrating telemedicine in the routine care practice. Participants agree that telemedicine can improve care for HF patients, but express that the promise of telemedicine has not yet been realised hampering a broader roll out of telemedicine in their routine practice. This observation is in line with the conclusion of a Dutch study performed already a decade ago.^23^ They concluded that the prior expectations of healthcare professionals in telemonitoring were not reflected in actual experience, which could hamper the adoption and scaling of telemedicine.

Our study shows that healthcare professionals experience challenges on several domains, that is, technological aspects of the intervention, the lack of guidance (inside and outside the hospital) as well as challenges about responsibility, liability and feelings of safety. In all these domains, professionals are continuously balancing the pros and cons of integrating telemedicine in daily practice. For example, professionals struggle with striking the right balance between surveillance of patients and taking the lead in decision making versus empowering them actively to take charge over their own health. While exerting control may enhance patients’ self-management skills to a lesser extent, patients are seeking for confirmation in their treatment from healthcare professionals. Additionally, integrating telemedicine into the EHR represents a technological challenge that holds promise for mitigating current workload burdens. Besides, other technical challenges are experienced as setting thresholds, and notifications lack clarity in urgency, which make telemedicine less efficient. The technical problems healthcare professionals face reflects their feelings of having less confidence in telemedicine. These perceived challenges may explain why only a minority of the patients use telemedicine to manage their disease. Healthcare professionals might be reluctant in offering telemedicine when they have less confidence in the patient due to patient characteristics such as age, digital skills and health literacy. Other studies confirm that patient characteristics are associated with receiving telemedicine, but are inconclusive about which characteristics (i.e. digital literacy, being a female, low level of SES).^14,24^ On the other hand, patients themselves might indicate that they not wish to use telemedicine because of feelings of safety and continuous confrontation with their disease, according to the interviewed healthcare professionals. Moulaei et al.^24^ conducted a mixed-methods study with patients formulating their reasons to choose either telemedicine or in-person visits. Reasons for selecting in-person visits instead of telemedicine included more accurate treatment, improved attention of the healthcare professional, safety aspects of patient privacy, lack of clear rules in the field of electronic disclosures of patient information.^24^ Both perspectives, professionals and patients, are important in understanding the low adoption of telemedicine in daily care.

The balance of empowering versus controlling the patient is related to feelings of confidence and safety in technology. This paradox was identified Mick and Fournier,^25^ which explained the impact of technology adoption on emotions. Their theory described that while technology can foster feelings of control, efficiency, and competence, it can also evoke contrasting outcomes as it enables individuals to unveil previously unrecognised needs, and increases reliance on technology, potentially leading to feelings of disorder, incompetence, inefficiency and isolation.^25,26^ The unrecognised needs for continuous monitoring was expressed in our study explaining that before the implementation of telemedicine, nurses did not had the possibility to monitor the HF patients at home which was perceived as no problem. Recognising this need and the perceived usefulness could actually foster the integration of telemedicine in HF care, but low feelings of confidence are a barrier to integrate telemedicine.

Multiple studies have evaluated the barriers and facilitators in the adoption and diffusion of new (digital) interventions in healthcare.^27–29^ The adoption of (technological) innovations is a result of a complex decision-making process.^30^ The theoretical model for telemedicine acceptance described that adoption and diffusion comprises three dimensions: the individual context (i.e. compatibility and attitude), the technological context (i.e. habits, perceived ease of use, perceived usefulness) and the organisational context (i.e. facilitators and subjective norm).^31^ These dimensions interact with each other, and influence the intention to provide (professional perspective) or use (patient perspective) telemedicine. From earlier evaluations of technological developments in healthcare, we know that innovations with substantial improvements in care are adopted earlier.^32,33^ Our study reveals that the perceived usefulness of telemedicine is ambiguity, resulting in a contradictory landscape of adoption and non-adoption of telemedicine. This could explain why only a minority of HF patient uses telemedicine. The respondents describe that not all individuals in the same team are in favour for using telemedicine. Whereas the providers’ acceptance is an important determinant of successful implementation of telemedicine.^34^ Telemedicine is experienced as additional care and not as substitution, resulting in less perceived usefulness and intention to use. In the study of Singhal et al.^10^, clinicians described a similar experience as in our study; time allocation changed when adopting telemedicine. Telemedicine consultation were perceived as more *efficient*, but integrating telemedicine requires more administrative time.^10^ However, a professional mention that it took time to make a change in daily practice to embrace telemedicine. Our findings are supported by previous studies about telemedicine in which they found challenges in several aspects of implementation, that is, lack of regulatory conditions, guidelines, workflows, which hamper the uptake of telemedicine.^35–37^ However, in these studies, less attention is paid about the challenges of emotional aspects in the adoption of telemedicine.

The integration of telemedicine in daily care practice asks for a shift in the professions of healthcare professionals. Our study exposes the need of structured care pathways to integrate telemedicine, which ask a change in the professional tasks. The needed revision of the occupational profiles is described in a recent article about revisiting the nursing metaparadigm. Johnson and Carrington^38^ argue that technology is foundational aspect to continue advancing nursing science and practice. Therefore the traditional nursing metaparadigm should change from four to five concepts: person, environment, health, nursing and technology.^38^ The integration of telemedicine asks for a patient centredness approach. Our findings support this approach, because the interviewed professionals explained that they aim for personalised telemedicine interventions. Healthcare professionals highlight that increased self-management skills could improve that patients feel more responsibility for their own health, and personalised telemedicine could be supportive. However, this again links to the paradox that nurse’s experience in their feelings of responsibility for the patient. Most research and theoretical frameworks focuses on what changes need to be made by healthcare professionals, in job descriptions, and organisational conditions, but it is important to add the patient perspective: ‘what does the patient need to use telemedicine’. This perspective requires for co-designing the telemedicine intervention, to ensure that telemedicine strategies fit the patients’ need, as well as the need of the professionals and supplier. Co-design is defined as ‘a process of collaborative design thinking or a joint inquiry and imagination where different participants associated with the design process work together to identify the problem, develop solutions, and evaluate those solutions’.^39,40^ Besides that co-design can support the development of a telemedicine tool, it can also improve the feelings of safety and confidence of nurses which is seen as a key aspect for the integration of telemedicine.

### Implications for practice

Our study identified three significant challenges that need to be addressed to facilitate the integration of telemedicine in HF care. The first major challenge is the clear definition of roles and responsibilities among patients, healthcare professionals, and telemedicine suppliers. A clear delineation of roles can enhance confidence among stakeholders. The second challenge is the establishment of structured care pathways that incorporate collaboration between patients, professionals, and suppliers. These pathways can support the clarification of roles and responsibilities. The third challenge is overcoming technological barriers through the co-design of telemedicine interventions. Co-design can increase the perceived usefulness of telemedicine and feelings of confidence from both healthcare professionals’ and patients’ perspectives. These findings might be interesting for telemedicine application in other medical conditions.

### Strengths and limitations

The main strength of our research is that we could explore in depth the experience of several types of telemedicine used in HF care management. To our knowledge, this is the first study that not only studied the technical and organisational barriers and facilitators to implement telemedicine, but also focused on aspects of doubts and reluctance of healthcare professionals to truly integrate telemedicine, despite its implementation at their HF outpatient clinic. The inclusion of multiple perspectives (i.e. nurses, nurse practitioners, cardiologists, team managers and manager’s digital health) enriched our findings, because it enabled us to identify experiences of telemedicine in different levels within the hospital, and sometimes contractionary perspectives and experiences. In addition, the interviews were held in several hospitals in the Netherlands with each hospital having its own telemedicine system, work processes, and implementation strategies. The inclusion of multiple hospitals with different telemedicine interventions and the inclusion of multiple perspectives supports the generalizability of our findings to other hospitals. However, the findings are less generalizable to other countries since only Dutch hospitals are included. Another strength is that the data collection and analysis were conducted by experienced researchers in qualitative research with backgrounds in nursing and/or clinical care. The main limitation in our study is that no patient perspectives and/or perspectives from telemedicine suppliers are included, whereas this could enrich the findings. Future studies should incorporate these perspectives. Nevertheless, we consider that for the aim of current study, we have included the right sample. Another limitation, is the potential for selection bias from our purposive sampling strategy. It is possible that more professionals with positive attitudes towards the use of telemedicine participated. However, we contend that despite their positive attitude against telemedicine, professionals were able of providing insightful reflections on current situation and critically expressing their experiences due to their extensive years of working with telemedicine. Finally, we did not pilot-tested the interview guide.

## Conclusion

Telemedicine is a promising intervention in the management of HF, but current healthcare systems and the telemedicine intervention itself is experienced as unmatured. The insights from this study can guide the integration of telemedicine demonstrating explicit attention is needed for the psychological aspects when implementing digital health. The next step in the integration of telemedicine current HF practices, is to start with designing a framework including clear care pathways within the hospital, collaboration with primary care, and clear agreements on tasks and responsibilities between healthcare professionals, patients and telemedicine suppliers. To substantially better incorporate the perspective of patients, co-design of the telemedicine platform and clinical care pathways is important. When this framework and the technological aspects of the intervention meet the needs of all involved parties concerned, there will be room to develop and grow feelings of responsibility, safety and confidence. Collaboration and regular evaluations between healthcare professionals, patients and telemedicine supplier are a prerequisite to integrate the multicomponent and multi-perspective intervention of telemedicine.

## Supplemental Material

sj-docx-1-dhj-10.1177_20552076241272570 - Supplemental material for Integrating telemedicine in routine heart failure management: Experiences of healthcare professionals – A qualitative study Supplemental material, sj-docx-1-dhj-10.1177_20552076241272570 for Integrating telemedicine in routine heart failure management: Experiences of healthcare professionals – A qualitative study by Jorna van Eijk, Jaap Trappenburg, Folkert W Asselbergs and Tiny Jaarsma in DIGITAL HEALTH

sj-docx-2-dhj-10.1177_20552076241272570 - Supplemental material for Integrating telemedicine in routine heart failure management: Experiences of healthcare professionals – A qualitative study Supplemental material, sj-docx-2-dhj-10.1177_20552076241272570 for Integrating telemedicine in routine heart failure management: Experiences of healthcare professionals – A qualitative study by Jorna van Eijk, Jaap Trappenburg, Folkert W Asselbergs and Tiny Jaarsma in DIGITAL HEALTH

sj-docx-3-dhj-10.1177_20552076241272570 - Supplemental material for Integrating telemedicine in routine heart failure management: Experiences of healthcare professionals – A qualitative study Supplemental material, sj-docx-3-dhj-10.1177_20552076241272570 for Integrating telemedicine in routine heart failure management: Experiences of healthcare professionals – A qualitative study by Jorna van Eijk, Jaap Trappenburg, Folkert W Asselbergs and Tiny Jaarsma in DIGITAL HEALTH
