# Supplementary material for: Integrating telemedicine in routine heart failure management: Experiences of healthcare professionals – A qualitative study
Source: Digit Health. 2024 Aug 28;10:20552076241272570. doi: 10.1177/20552076241272570 (PMC11363038; doi:10.1177/20552076241272570)
Supplement: sj-docx-2-dhj-10.1177_20552076241272570 - Supplemental material for Integrating telemedicine in routine heart failure management: Experiences of healthcare professionals – A qualitative study [file sj-docx-2-dhj-10.1177_20552076241272570.docx]

**Supplementary file 2 – Interview guide**

*Interview guide for nurses, nurse practitioners and cardiologists*

## Introduction

#### What do we mean by telemedicine?

With telemedicine, we mean the provision of care at a distance. The healthcare provider and the patient are not physically present with each other at that moment. Telemedicine can consist of telemonitoring or tele-education (according to the Collaboration Agreement and Quality Criteria for Telemedicine).

- **Telemonitoring** involves monitoring heart failure symptoms and relevant parameters used to support patients and healthcare providers in the treatment of heart failure.
- **Tele-education** is a service that allows the patient/user to receive remote education. This remote learning can be done through various techniques such as television or the internet. Education can also be provided in various (digital) ways.

### Background Information: Use of Telemedicine

1. Does your hospital offer telemedicine to outpatient heart failure patients?

- Yes, we offer telemedicine.
  1. Why do you offer telemedicine?
     - *Possibilities: monitoring, education, self-care/self-management, consultation, cost reduction, reduction in hospital admissions, improvement in quality of life, reducing workload.*
- No, we do not offer telemedicine.
  - 1. Why don't you offer telemedicine? (How strong is the influence of [x] in this?)
       - *Possibilities: no patient/clinician demand, cost, evidence, user-unfriendly technology, no connection to EHR, HF care pathway is different, time, shortage of healthcare professionals, significant investment compared to patients, legal/privacy concerns, others.*
    2. Have you considered using telemedicine for HF patients?
       - What prompted you to consider it but not proceed?
    3. Which professionals were involved in this decision?
- No, but we plan to start telemedicine.

1. Why are you planning to start telemedicine?
   - *Possibilities: patient/clinician desire, guidelines, COVID-19, funding, others.*
2. To what extent did COVID-19 influence this decision?
3. Which professionals are involved in this decision?

- No, we stopped telemedicine.

1. Why did you stop telemedicine?
   - *Possibilities: no patient demand, didn't meet expectations, more time, moderate positive effects, cost, lack of staff, others.*
2. Who were involved in this decision? (Professionals)

## Users of Telemedicine

*From this point on, the conversation continues for users of telemedicine.*

1. Which system do you use for telemedicine?

- Luscii
- Sananet/SanaCoach
- Motiva (Philips)
- 24Care
- Hartwacht
- CardioMEMS
- Empower
- Other, namely: ...

1. Why do you use telemedicine for heart failure patients?
   - Why is telemedicine used for [goal X]?
2. *Functionalities: monitoring, education, self-care/self-management, consultation.*
3. *Endpoints: cost reduction, reduction in hospital admissions, improvement in quality of life, reducing workload for healthcare professionals.*
4. *Others, namely: ...*

### Offering Telemedicine

1. What kind of patients come to your heart failure clinic?
2. *Severity, age, duration of illness.*
3. Which heart failure patients at your clinic are eligible for telemedicine?

- What factors are considered in the decision to use telemedicine? (Is this everything?)
  - 1. *Telemedicine guideline: Chronic HF, recently diagnosed, readmission due to exacerbation, anxious/uncertain/depressive patients, assistance in early detection of deterioration.*
    2. *Characteristics: caregiver network, patient/donor digital skills, language proficiency, patient mobility, internet connection, distance to hospital, others.*
- Does the severity of HF play a role in initiating telemedicine?
  - Yes
  - No
- At which NYHA class is telemedicine used?
  - NYHA 1
  - NYHA 2
  - NYHA 3
  - NYHA 4
- Why this NYHA class?

1. Which heart failure patients at your clinic are not eligible for telemedicine?

- Why are these patients not eligible?
  1. *Telemedicine guideline: Chronic HF, recently diagnosed, readmission due to exacerbation, anxious/uncertain/depressive patients, assistance in early detection of deterioration.*
  2. *Characteristics: caregiver network, patient/donor digital skills, language proficiency, patient mobility, internet connection, distance to hospital, others.*

1. When is telemedicine first considered for the HF patient? (multiple answers possible)

- For every newly diagnosed heart failure patient
- After a hospital admission
- For patients attending the HF clinic (regardless of previous hospital admission)
- After a heart failure exacerbation (patient is already known to have heart failure)
  1. When is telemedicine offered after an exacerbation?
     - After 1 exacerbation
     - After ... exacerbations
     - If the patient has more than ... exacerbations
  2. Does the time period in which the patient has an exacerbation play a role in offering telemedicine?
     - Yes, when ... exacerbations in ... weeks
     - No
     - In the titration phase
  3. Is telemedicine offered during the titration phase?
     - Yes, every time there is a change in medication
     - Yes, only when the patient is newly diagnosed with the disease
     - Sometimes, depending on the situation, ease of titration, patient's distance, pandemic, etc.
     - No
  4. For which medication?
- In the stable phase of HF
  1. Is telemedicine offered in every stable phase of heart failure?
     - Yes
     - No
- Others, namely: ...

1. Which healthcare professionals are involved in determining whether or not to use telemedicine for the patient? (multiple answers possible)

- Cardiologist
- Heart failure nurse
- Nurse Specialist / Physician Assistant
- General Practitioner
- Home care worker
- Practice Nurse
- Others, namely: ...

### Stopping/On-Off Principle of Telemedicine

1. Is telemedicine offered multiple times in a heart failure care trajectory? (on/off/on/off principle)

- Yes
  1. How is this offered?
  2. For which patients?
  3. What determines the on/off/on/off principle?
  4. Is the equipment removed?
- No

1. When is telemedicine stopped?
   1. *Patient request, death, healthcare professional's assessment, disease severity, treatment phase, established time period*

- Does NYHA class play a role in this?

### Modules of the Telemedicine System

1. On what systems/via what digital channels can the patient receive telemedicine? *(multiple answers possible)*

- Application (App) on a mobile phone (smartphone)
- Computer
- Television
- Watch (smartwatch)
- iPad/tablet
- Others, namely: ...

#### Telemonitoring

1. Does your telemedicine system include telemonitoring (e.g., vital signs, nutrition, medication, symptoms)?

- Yes
  1. What is the main reason for using telemonitoring?
     - *Early detection of exacerbation, titration, longitudinal follow-up, self-management, others*
- No

1. What form of telemonitoring do you use?
   - Non-invasive
   - Invasive
   - Non-invasive and invasive
2. Which measurements are monitored in telemonitoring?

- Weight
- Blood pressure
- Heart rate
- Heart rhythm (irregular, regular)
- Temperature
- Saturation
- Intake (e.g., in the context of sodium restriction)
- Fluid intake
- Medication
- Heart failure-related symptoms
  1. What symptoms do you monitor?
     - *Fatigue, decreased appetite, shortness of breath, swollen legs and ankles, cold hands and feet, a full feeling in the upper abdomen, palpitations, nocturia, constipation, tickling cough, restless sleep, memory and concentration problems, dizziness*
     - *Other, namely: ….*
- Other, namely: ....

1. What equipment has the patient received for telemonitoring? *(multiple answers possible)*

- No equipment
- Patient uses their own equipment
  1. What equipment?
- Scale
- Bluetooth-enabled scale
- Blood pressure monitor
- Bluetooth-enabled blood pressure monitor
- Pulse oximeter (Saturatiemeter)
- Bluetooth-enabled pulse oximeter
- ECG device
- CardioMEMS
- Other, namely: ....

1. Can a patient transmit monitored functions via the telemonitoring system to the healthcare provider?

- Yes
  1. When can the patient transmit these monitored functions? (time of day)
- *Possible options: 24/7, office hours, weekend, evening, night, set times, other, namely*
  1. How does the patient transmit the monitored functions to the healthcare professional? (communication)
     - *Possible options: letter, phone call, video call, chat, SMS, email, via applications, other*
- No

1. Does the healthcare professional receive a notification when monitored functions are entered into the telemonitoring system?

- Yes

1. When does the healthcare professional receive a notification?
   - *Possible options: always, when threshold values are exceeded, other*
2. Where does the healthcare professional receive the notification?
   - *Possible options: Electronic Patient Record (EPD), standalone applications, email inbox, other*
3. Are the notifications to the healthcare professional always received in the same way, or can severity and exceeding of threshold values play a role?
   - Yes
   - No
     1. What is the difference?
4. Which healthcare professional receives results/values?
   - *Possible options: cardiologist, heart failure nurse, nurse practitioner, physician assistant, general practitioner (GP), practice nurse, home care worker, other*
5. Are there ever erroneous notifications?
   - *How do you handle them?*

- No

1. How often does the healthcare professional evaluate the values, questions, outcomes filled in by the patient in the telemonitoring system?
   1. *Per week: ....*
   2. *Per day: ....*
   3. *Per patient: ....*
2. Can a healthcare professional respond to the received results/values of the monitored functions?

- Yes

1. Which healthcare professional responds to the received results/values?
   - *Possible options: cardiologist, heart failure nurse, nurse practitioner, physician assistant, general practitioner (GP), practice nurse, home care worker, other*
2. When can the healthcare professional respond to these monitored functions? (time of day)
   - *Possible options: 24/7, office hours, weekend, evening, night, set times, other*
3. How does the healthcare professional respond to the monitored functions?
   - *Possible options: letter, phone call, video call, chat, SMS, email, via applications, other*
4. Does the patient receive a notification/message if the healthcare professional has sent a message/action to the patient?

- No

1. Can threshold values be set for the monitored functions in the telemonitoring system?

- No
- Yes, general threshold values (not patient-specific)
  1. What are the threshold values based on? / How are the threshold values formulated?
- Yes, patient-specific threshold values
  1. Can the threshold values be adjusted per patient, per situation, per moment?
     - Yes
     - No
  2. What are the threshold values based on? / How are the threshold values formulated?

1. Is there always contact between the patient and the healthcare professional for the monitored functions transmitted via the telemonitoring system?

- Yes
  1. Is the contact direct or indirect (store-and-forward) between the patient and the healthcare professional?
     - *Direct: The patient and healthcare professional are using the system simultaneously, allowing for direct contact via one of the techniques provided by the telemonitoring system.*
     - *Indirect: The patient and healthcare professional have asynchronous contact with each other. The patient/healthcare professional sends their information to the recipient. The recipient reads the message later and responds to the sender.*
     - *Combination of direct and indirect contact*
- No
  1. When is there contact between the patient and healthcare professional?

1. What actions does the healthcare professional take with the received monitored functions?
   - 1. How do you initiate these actions?
        - *Possible options: letter, phone call, video call, chat, SMS, email, via applications, other*
     2. Does the telemonitoring system provide automatically generated advice to the patient when they enter/select data in a module in the telemonitoring system?
        - *Yes*
        - *No*
2. What is your experience with the telemonitoring provided by the telemonitoring system?

#### Education and self-care

1. Does your telemonitoring system include education and self-care?

- Yes
  - 1. Does the education module need to be actively activated for each patient by the healthcare professional? (Note: Can the telemonitoring system also exist without the education module?)
    - Yes
    - No
    1. What does the education cover?
       - General medical information about the HF condition
       - Information about treatment
       - Prevention of symptoms/exacerbations
       - Medication use
       - Fluid and dietary management
       - Physical activity
       - Other, namely: ...
    2. Do you actively refer the patient to the education module in the telemonitoring system? (Note: Do you discuss this topic in your consultation?)
    - Yes
    - No
  1. Is the education module the same for every patient?
  2. Which self-care components are included in the telemonitoring system?
- Lifestyle interventions,
  - Which behaviors/lifestyle interventions are included?
    - *Possible answers: exercise, nutrition, stress management*
- Psychological well-being: information, knowledge, tools
- Patient's medication overview
- Medication explanation (medication glossary)
- Medication intake reminder as per prescription
- Referral to external sources and links related to heart failure
- Knowledge quiz/ability to test knowledge about heart failure
- Monitoring
- Self-detection and the ability to anticipate/adjust medication (e.g., detecting weight gain through monitoring and then taking and recording medication [diuretics])
- Other, namely: ...
- No

1. To what extent does the patient make decisions based on the telemonitoring system?
   - To what extent is there a focus on the patient actively taking actions and anticipating detection?
2. To what extent does the telemonitoring system promote the patient's self-care? / What is the effect of this module on the patient's self-care (skills)?
3. What are your experiences with the built-in self-care and education components in the telemonitoring system?

#### Heart Failure Community

1. Can the patient use the telemonitoring system to get in touch with peers (for example, a heart failure community)?

- Yes, always
- Yes, but this module needs to be activated
  1. How is the heart failure community organized?
     - *Local (hospital)*
     - *Regional*
     - *National*
- No, this is not possible

### Analog Telemonitoring (via telephone)

1. Is there telephone contact with heart failure patients (from the hospital)?

- Yes
  - 1. Who initiates telephone contact with these patients?
       - *Possible options: cardiologist, heart failure nurse, nurse practitioner (NP), physician assistant (PA), general practitioner (GP), practice nurse (POH), home care worker, other*
    2. When is telephone contact made with the patient?
    3. For what purpose is telephone contact made?
    4. How often is telephone contact sought with the patient?
    5. Which patients receive telephone guidance?
    6. Why these patients?
- No

1. Can the patient initiate telephone contact with the heart failure clinic (from the patient's side)?

- Yes
  - 1. When can/may the patient initiate telephone contact?
    2. Who does the patient have telephone contact with?
       - *Possible options: cardiologist, heart failure nurse, nurse practitioner (NP), physician assistant (PA), general practitioner (GP), practice nurse (POH), home care worker, other*
- No

1. Can a heart failure patient or healthcare professional seek contact in any other way?
   - Through which channel does this contact occur?
     - - *Possible answer: patient portal*
   - When does this contact occur via this channel?
2. What is your experience with telemonitoring via telephone contact?

### External Influences on the Use of Telemonitoring

#### Evaluation

1. Is the telemonitoring system evaluated (experiences, application in HF care, impact on objectives) among healthcare professionals?

- Yes
- No

1. What is the influence of telemonitoring on the patient population that visits the (regular) heart failure clinic?

- Which heart failure patients are these? (characteristics of HF, personal characteristics)

1. Is the telemonitoring system evaluated (experiences, application in their disease, usage) between the healthcare professional and the patient?

- Yes
- No
  - 1. Are expectations discussed with the patient regarding the use of telemonitoring?
    2. What do patients indicate? What feedback do you receive?
    3. How is the telemonitoring experience perceived?

#### Experience

1. Do you believe that telemonitoring in your center has led to effectiveness/efficiency?
   - - - Yes
         1. On which outcomes?
         2. Has this been objectively assessed?
       - No
         1. What makes you say that?
2. Would you recommend your system to other organizations?
   - - - Why or why not?
3. What would you like to change/improve about your system?
4. If you could choose a telemonitoring intervention again, what would it look like?

#### COVID-19

1. Does COVID-19 have an impact on the use of telemonitoring in your heart failure patient population? (multiple answers possible)
   - - - Yes, telemonitoring is used for more patients as an intervention.
       - Yes, telemonitoring is used less frequently for patients as an intervention.
       - Yes, telemonitoring is used differently by patients who were already using this intervention before COVID-19.
     1. Which techniques of the telemonitoring system are used differently since COVID-19?
        - *Possible answers: phone calls, video calls, chat, email, SMS, chatbot, other.*
     2. Which modules of the telemonitoring system are used differently since COVID-19?
        - *Possible answers: telemonitoring, education, self-care/self-management, heart failure community, other.*
     3. What do you think of this development?

- No, there is no difference in the use of telemonitoring before, during, and in the current COVID-19 period.

## Time Investment in Telemonitoring vs. No Telemonitoring in Heart Failure Care (HTA)

1. How much time do you, on average, spend on a heart failure clinic consultation without telemonitoring?

*... minutes / hours*

1. How much time do you, on average, spend on a heart failure clinic consultation with telemonitoring?

*... minutes / hours*

1. How much time does it take to explain telemonitoring to a heart failure patient (for the initial setup)?

*... minutes / hours*

1. How much time do you, on average, spend per week on providing telemonitoring? (this includes monitoring digital statuses transmitted through the telemonitoring system, contacting the patient as needed, evaluating values, etc.)

*... minutes / hours*

*Interview guide for team managers and managers digital health*

| Topic | Questions |
| --- | --- |
| Background information and aim | - When did you first start using telemedicine? - Why do you use telemedicine ? - For what purpose is telemedicine used? Are there any other purposes? |
| Telemedicine supplier | - Who is the supplier? - Do you have experiences with other suppliers/telemedicine application? Did consider other suppliers? - What was decisive in choosing this supplier (compared to possible other suppliers)? - What were important elements in the choice of supplier? - How does the choice of supplier relate to your goal for telemedicine? - Who were/are involved in the choice of supplier/TB system? - What was their role? |
| Hospital organisation | - What is the vision of your hospital (board) regarding the use of telemedicine? - How does the vision of the cardiology department regarding telemedicine relate to the hospital-wide vision on telemedicine ? - Is telemedicine also used in other specialties? If so, which ones? |
| Heart failure care (vision/aim) | - Why do you have telemedicine in heart failure care? - Was there a need of professionals to implement telemedicine? - How does the vision towards telemedicine relate to the aim of telemedicine ? |
| Proof of evidence | - What was decisive for the cardiology department in the decision of use telemedicine? - Does the use of telemedicine depend on: guidelines, literature, specialty group, professional association, patient federation, hospital policy etc.? - How does the prevailing view on telemedicine affect your reasoning for using telemedicine? |
| Implementation | - How was the process of implementation of telemedicine in heart failure care? - Who were involved? - Which role had these professionals? - What were barriers to implementation? - What were promoting factors to implementation? |
| Preconditions | - What was needed form the hospital board to achieve implementation of telemedicine? - What was needed from the Department of Cardiology to achieve implementation of telemedicine? - What was required from the healthcare professional to achieve implementation? - To what extent was there a shift of tasks in healthcare professionals needed to implement telemedicine? |
| Health Insurance Company | - What is the role of the health insurance company in the implementation of telemedicine? - How are the costs for telemedicine claimed? |
| Values | - What is the value of using telemedicine for:   - The hospital   - Healthcare professionals   - Patients |
| Evaluation | - What were the expectations when using telemedicine in heart failure care? - Are goals evaluated? - What is being evaluated? - Who are involved in these evaluations? - How often is the evaluation? - Does evaluation of the telemedicine application take place with patients? - What is being evaluated with the patient? - How often is the evaluation? - Do you think that telemedicine has led to effectiveness and/or efficiency? On what outcomes? - What is your future vision regarding telemedicine in heart failure care? |
| Primary care | - Do transmural care pathways including agreements about telemedicine exist? - Is primary care involved in telemedicine? Why? |
| Recommendations and improvements | - Would you recommend your telemedicine application? Why? - What would you change/improve in the telemedicine application? |
