# Supplementary material for: Integrating telemedicine in routine heart failure management: Experiences of healthcare professionals – A qualitative study
Source: Digit Health. 2024 Aug 28;10:20552076241272570. doi: 10.1177/20552076241272570 (PMC11363038; doi:10.1177/20552076241272570)
Supplement: sj-docx-3-dhj-10.1177_20552076241272570 - Supplemental material for Integrating telemedicine in routine heart failure management: Experiences of healthcare professionals – A qualitative study [file sj-docx-3-dhj-10.1177_20552076241272570.docx]

**Supplementary file 3 – clustering of codes**

Overview of the codes and subcodes derived from the interviews.

| **Code** | **Subcode** | **Detailed subcode** |
| --- | --- | --- |
| **Purpose of telemedicine** | Primary care |  |
|  | Collaboration |  |
|  | Detection |  |
|  | Patient-friendly |  |
|  | Titration |  |
|  | Volume |  |
|  | Workload |  |
|  | Self-management |  |
|  | Reducing hospital visits |  |
|  | Cost-effectiveness |  |
|  | Innovative |  |
|  | Quality of care |  |
| **Evaluation of the system** | Evaluation | *Patients* |
|  |  | *Employees* |
|  | Identifying other health problems by chance | *Trends* |
|  |  | *Diseases* |
|  | Problems |  |
|  | (False sense of) safety |  |
| **Features of the telemedicine intervention** | Communication | *Messages* |
|  |  | *Thresholds* |
|  |  | *Notifications* |
|  | Peer support |  |
|  | Measurements | *Blood pressure* |
|  |  | *Depression* |
|  |  | *Weight* |
|  |  | *Heart rate* |
|  |  | *Lifestyle* |
|  |  | *HF symptoms* |
|  | Preparation for outpatient clinic |  |
|  | Stopping with telemedicine | *Feasibility* |
|  |  | *Palliative* |
|  | Information |  |
|  | Education | *Physical* |
|  |  | *Telemedicine* |
|  | Adjustments |  |
|  | Self-management |  |
| **Usability of the telemedicine intervention** | Patient |  |
|  | Healthcare professionals | *External system* |
|  |  | *Connection with EHR* |
| **Implementation** | General |  |
|  | Stakeholders |  |
|  | De-implementation |  |
|  | Team involved in telemedicine |  |
|  | Team characteristics |  |
| **Organisation** | Continuity |  |
|  | Workflow |  |
|  | Outpatient clinic policy |  |
|  | Hospital | *Problems* |
|  | Monitoring center |  |
|  | Primary care |  |
|  | Hospital policy |  |
|  | COVID-19 |  |
|  | Vision | *Unknown* |
|  |  | *Cardiology* |
|  |  | *Patient* |
|  | Health insurance company | *Name* |
|  |  | *Function* |
| **Patient characteristics** | Comorbidity |  |
|  | HF diagnosis |  |
|  | Digital skills |  |
|  | Personal choice |  |
|  | No telemedicine |  |
|  | Everyone |  |
|  | Unstable or stable HF patients |  |
|  | Lifestyle |  |
|  | Age |  |
|  | Caregiver |  |
|  | Motivation |  |
|  | Socio-economic status |  |
|  | Titration |  |
|  | Residency |  |
| **Requirements for telemedicine implementation** | Evidence |  |
|  | Funding |  |
|  | ICT |  |
|  | Knowledge |  |
|  | Personnel |  |
|  | Politics |  |
|  | protocol |  |
|  | Guidelines |  |
|  | System |  |
|  | Time |  |
|  | Workload |  |
|  | Profit |  |
| **Role of the healthcare professionals** | Task shifting |  |
|  | Responsibility |  |
| **Telemedicine tool** | Equipment |  |
|  | Reasons for choosing type of telemedicine tool | *General* |
|  |  | *No alternative* |
|  |  | *Evaluation* |
|  |  | *Extra system* |
|  |  | *Integration* |
|  |  | *Collaboration* |
|  |  | *Based on research project* |
|  | Content |  |
|  | Protocol |  |
|  | System |  |
| **Future** | Policy |  |
|  | Heart failure |  |
|  | Telemedicine |  |
| **Invasive telemedicine** |  |  |
| **Analogue monitoring** |  |  |
| **Health Technology Assessment** |  |  |
